# Ten Simple Rules for Effective Computational Research

**DOI:** 10.1371/journal.pcbi.1003506

**Published:** 2014-03-27

**Authors:** James M. Osborne, Miguel O. Bernabeu, Maria Bruna, Ben Calderhead, Jonathan Cooper, Neil Dalchau, Sara-Jane Dunn, Alexander G. Fletcher, Robin Freeman, Derek Groen, Bernhard Knapp, Greg J. McInerny, Gary R. Mirams, Joe Pitt-Francis, Biswa Sengupta, David W. Wright, Christian A. Yates, David J. Gavaghan, Stephen Emmott, Charlotte Deane

**Affiliations:** 1 Computational Biology Group, Department of Computer Science, University of Oxford, Wolfson Building, Oxford, United Kingdom; 2 Computational Science Laboratory, Microsoft Research, Cambridge, United Kingdom; 3 CoMPLEX, Mathematical and Physical Sciences, University College London, Physics Building, London, United Kingdom; 4 Centre for Computational Science, Department of Chemistry, University College London, London, United Kingdom; 5 Wolfson Centre for Mathematical Biology, Mathematical Institute, University of Oxford, Andrew Wiles Building, Radcliffe Observatory Quarter, Oxford, United Kingdom; 6 Department of Statistics, University of Oxford, Oxford, United Kingdom; 7 The Wellcome Trust Centre for Neuroimaging, Institute of Neurology, University College London, London, United Kingdom; University of California San Diego, United States of America

In order to attempt to understand the complexity inherent in nature, mathematical, statistical and computational techniques are increasingly being employed in the life sciences. In particular, the use and development of software tools is becoming vital for investigating scientific hypotheses, and a wide range of scientists are finding software development playing a more central role in their day-to-day research. In fields such as biology and ecology, there has been a noticeable trend towards the use of quantitative methods for both making sense of ever-increasing amounts of data [1] and building or selecting models [2].

As Research Fellows of the “2020 Science” project (http://www.2020science.net), funded jointly by the EPSRC (Engineering and Physical Sciences Research Council) and Microsoft Research, we have firsthand experience of the challenges associated with carrying out multidisciplinary computation-based science [3]–[5]. In this paper we offer a jargon-free guide to best practice when developing and using software for scientific research. While many guides to software development exist, they are often aimed at computer scientists [6] or concentrate on large open-source projects [7]; the present guide is aimed specifically at the vast majority of scientific researchers: those without formal training in computer science. We present our ten simple rules with the aim of enabling scientists to be more effective in undertaking research and therefore maximise the impact of this research within the scientific community. While these rules are described individually, collectively they form a single vision for how to approach the practical side of computational science.

Our rules are presented in roughly the chronological order in which they should be undertaken, beginning with things that, as a computational scientist, you should do *before* you even think about writing any code. For each rule, guides on getting started, links to relevant tutorials, and further reading are provided in the supplementary material (Text S1).

## Rule 1: Look Before You Leap

One of the key considerations in the development of any method, computational or otherwise, is whether it has previously been approached by someone else. A growing wealth of software toolboxes and libraries exist to tackle many problems. However, assessing the range and quality of what is available can be hard, especially when addressing nontraditional problems. A simple but often-overlooked approach is to conduct a software literature review to ascertain what software is available and has been successfully employed. Software repositories (e.g., GitHub, https://github.com/, and SourceForge, http://sourceforge.net/) are a good place to begin a review. Furthermore, engaging with the network of researchers surrounding your own is invaluable; see [8] and [9] for advice on this. If your coworkers write software in the same language or use particular toolboxes, you may be able to consult their expertise in order to accelerate and provide support for your work.

## Rule 2: Develop a Prototype First

Before writing any code, it is imperative to clarify what you are trying to implement: what functionality do you require, and what interfaces do you need? When implementing your latest developments, you should first begin by considering a prototype (i.e., a simplified version of the full system or algorithm) to gain insight and to guide the next steps. This is equally relevant whether building on existing code or starting from scratch. By prototyping new functionality and building code up incrementally, you can check that each element of your code operates as expected (and each incremental development can be tested; see Rule 8). Breaking your problem up into smaller elements like this will also help to provide structure to your code and will make it much easier when you subsequently need to extend it. From a practical point of view, it will usually be easier to prototype mathematical and statistical methods in a “higher-level” language, for example Matlab, R, or Python. Although these languages can be slower to run than optimized code in a “lower-level” language, their straightforward nature, built-in functionality, and available libraries mean that you will spend less time expressing your ideas in code and searching for bugs.

## Rule 3: Make Your Code Understandable to Others (and Yourself)

When revising or adapting existing code, the absence of documentation and comments can result in errors and time drains. Such documentation not only makes your code more understandable to others but also to your future self (put simply, the code tells you “how”, the comments tell you “why”). The program code itself can be made more understandable by using meaningful variable names and formatting the code consistently. While commenting and documentation is often neglected when faced with deadlines, developing and maintaining a standardised way of commenting your code will be of great benefit. As well as low-level documentation in the code, you should maintain a record of the “big picture” functionality (i.e., interconnectivity of components and input/output formats). This could take the form of a high-level diagram or description of the system, whether by hand on paper, in verbose code comments, or using standardized approaches such as UML (Unified Modelling Language) (see Text S1). When you are reviewing your code for documentation you should actively seek ways to break it up into modules. This not only aids structure and readability but also avoids the error-prone and tedious task of debugging and updating two (or more) copies of the same code. As a rule of thumb, if you write the same code twice, it should become a function, subroutine, or method.

## Rule 4: Don't Underestimate the Complexity of Your Task

When developing your code, you should keep a record of your work. This could be in the form of a “logbook” file or a paper notebook where you store commonly used commands and other notes; another good option is an online tool such as Evernote (http://evernote.com/). You will often find that you have to choose between spending a long time doing a task by hand and possibly spending longer learning how to automate it. In order to automate the task, you will probably need to learn how to use some basic tools such as text editors or scripting languages. Don't be tempted to think, “This is just a one-off, I'll get on with it;” it won't be. You will find bugs, wish to change a parameter, or need to alter a figure slightly, and you will eventually have to repeat the whole process. Even if you are *certain* that it really is a one-off task, use your “logbook” and keep a record of the list of commands you used, since this is the first step towards automating the task if and when the time comes. However, it is not appropriate to automate everything, and you need to find a good balance, automating opportunistically, taking the expected time and cost into account. A good rule to follow is “the rule of three:” once you have had to do the same thing twice already, automate it.

## Rule 5: Understand the Mathematical, Numerical, and Computational Methods Underpinning Your Work

When solving any computational model, you should always ensure that you are using the appropriate numerical method for your problem, and that any constraints and conditions are satisfied. A basic understanding of numerical analysis and, in particular, the concepts of rate of convergence, order, and stability of numerical methods will pay dividends. Care should also be taken to ensure that any assumptions made in the derivation of the underlying mathematical models or methods (e.g., having a sufficiently large number of objects to permit a continuum approximation) hold for all system states of interest. You should consult the relevant literature (and communities) that explains these methods and their advantages and/or disadvantages and not steam ahead without first gaining an understanding of which methods are appropriate. By fully understanding the mathematical and numerical methods being used, you can be confident that your results reflect the true behaviour of the underlying model and are not numerical or computational artefacts.

## Rule 6: Use Pictures: They Really Are Worth a Thousand Words

Visualisation and graphics are fundamental to developing, understanding, and testing hypotheses, and are indispensable for verifying and validating computational methods (e.g., revealing correlations, covariation, position, structure, flows, orientation, anomalies, and outliers). So, from day one, spend time developing the visual components of your work. Learn, develop and use visualisation software and tools to ensure that you understand your research outputs and can effectively communicate your findings. You may well need to develop novel visualisations for your work, but keep the basic figures. You needed them to understand your results, model, and implementation, and so will anyone else. You should ensure that your visualisation algorithms can be executed separately so that they can be reused by you and others (for the same and different tasks) and refined for other formats (e.g., publications, presentations, and websites). In reality, all scientists could be better educated in design, so any investment will be rewarded, especially by receiving feedback on visualization from users.

## Rule 7: Version Control *Everything*

Version control systems (VCSs) offer an easy way to store and back up not only the current version of your code that you are working on but also every previous version of the code (in what's known as a repository). This not only saves you from having to keep multiple copies of the same file but also allows you to “roll back” to an older “working” version of the code if things go wrong. VCSs also allow you to share material between multiple machines, operating systems, and more importantly, users in a simple and robust manner. Two of the most popular VCSs are Subversion (http://subversion.apache.org) and Git (http://www.github.com), both of which offer many advanced features for managing your code. Cloud storage such as Dropbox (http://www.dropbox.com) and SkyDrive (http://www.skydrive.live.com) offer basic file sharing and backup facilities; however, they don't offer the code management features of true VCSs, so the effort put in to learning a VCS is well worth it (see Text S1 for guides on getting started with VCSs). While the primary use of version control is to manage the development and distribution of code, many other collaborative endeavours can be stored in a version control repository. In particular, using version control tools while preparing publications can save time and effort, especially when dealing with input from multiple authors. For example, contributions to this manuscript were managed using a VCS.

## Rule 8: Test *Everything*

Any non-trivial computer program will have bugs when first written, often subtle ones that are hard to detect, which may lead to incorrect results. Indeed, in extreme cases this has caused high-profile retractions of papers [10]. Simple tests that the software behaviour matches expectations are essential for ensuring robust results, minimising the presence of bugs, and gaining confidence in your code (for you and others). As a result of the time pressures inherent in academia, often software testing is performed manually in an *ad hoc* manner, to determine whether results “look roughly right” [11]. However, a systematic approach to testing pays dividends. You should learn how to test effectively to avoid the illusion of reliability. For example, compare low-level routines against analytical or prototype solutions (see Rule 2) or experimental data and consider “corner cases” and both branches of “if” statements. Get the computer to run tests for you automatically and alert you to problems, using a suitable testing framework (see Text S1). Ideally this should be tied to a version control system (see Rule 7) so that tests are run automatically whenever new code is committed to the repository. A useful rule is to turn bugs you fix into new tests to avoid them recurring. Testing gives you the confidence to modify your code without worrying that you are breaking it. Testing can also provide a means for reproducing results of published papers. By setting up a test comparing against published values, you can easily find out when fixing a newly identified bug changes published results.

## Rule 9: Share *Everything*

Just as it is a common practice to publish your research findings in peer-reviewed journals, if an important part of your research involves developing new software tools and/or collecting new data, you should consider sharing these [7]. Based on our collective experience, we advocate an open approach of sharing source code, data, and results as freely as possible. You should ask yourself, “Why not share?” If the answer is, “I am worried that people would find mistakes in it,” then, as a scientist, this should be the strongest argument in favour of sharing it! The provision of such resources openly provides the means to replicate, reproduce, and examine newly developed methods and techniques. Open sharing not only facilitates the scientific enterprise through replication, validation, and error checking, but also deters fraud and malpractice through transparency. It is our opinion that the many arguments in favour of openly sharing code, data, and results far outweigh any against. In many modern computational analyses, the source code represents a readable, executable methodology of the research in question. Sharing is the key to a sustainable future for computational science, and publishers are beginning to require it, with some considering reviewing the software used to generate results [12].

## Rule 10: Keep Going!

Our advice arises from our collective experience, and we continue to strive to obey these rules in our work. Scientists have a wide variety of demands on their time (researching, writing papers [13], teaching [14], applying for grants, administration, etc.) and have to make the most of limited resources. Becoming more technically effective can seem daunting without strategies for making progress and keeping motivated. So, prioritise in a way that suits you and your projects and career aspirations. One strategy is to implement another of these rules each time you start a new project, to build a growing repertoire rather than trying to do everything at once. Take every opportunity to teach and help others to do what you have learnt.

## Supporting Information

Text S1 Supplementary material for paper. Includes guides for getting started with each rule, along with references to useful links and further reading.
(PDF)
