# Supplementary material for: Ten Simple Rules for Effective Computational Research
Source: PLoS Comput Biol. 2014 Mar 27;10(3):e1003506. doi: 10.1371/journal.pcbi.1003506 (PMC3967918; doi:10.1371/journal.pcbi.1003506)
Supplement: Text S1 — Supplementary material for paper. Includes guides for getting started with each rule, along with references to useful links and further reading. (PDF) [file pcbi.1003506.s001.pdf]

Ten Simple Rules for Effective Computational Research: Supplementary Material

Introduction

This supplementary material, for the paper ‘Ten Simple Rules for Effective Computational Research’, is designed to provide more detailed guides to getting started with the rules specified in the paper.

It is broken up into sections, which map onto the rules. Each section contains the following information: a short guide/tips for ‘**Getting started**’; ‘**Useful links**’ to articles and websites of interest for the general reader to help with getting started; and a list of more in-depth references for ‘**Further reading**’.

While we have strived to be as complete as possible we could not hope to provide an exhaustive list of software and links. From our varied interests we have compiled a set of links and references that should serve as a solid starting point.

Rule 1: Look before you leap

Getting started

- Talk to other people in your group to discover the sorts of software they use and whether you can make use of it.
- Be aware of licensing restrictions on software you want to use; the Open Source Initiative (http://opensource.org/licenses/category) and OSS Watch (http://www.oss-watch.ac.uk/) are good websites to consult.
- An article on conducting systematic literature reviews in software engineering:
    - P. Brereton, B. A. Kitchenham, D. Budgen, M. Turner, and M. Khalil. Lessons from applying the systematic literature review process within the software engineering domain, Journal of Systems and Software, 2007, 80(4), 571–583, http://dx.doi.org/10.1016/j.jss.2006.07.009

Useful links

- Wikipedia provides a list of some established software repositories: http://en.wikipedia.org/wiki/Software_repository
- GitHub (https://github.com/) and SourceForge (http://sourceforge.net/) are two of the most popular software repositories and contain all types of open source software including scientific tools.
- MATLAB (http://www.mathworks.co.uk/products/matlab/) is a widely-used scientific computing environment and programming language developed by MathWorks. While MATLAB itself is commercial software, there is a wide variety of open source code written for MATLAB available online. MATLAB Central is one such repository and is a great place to find MATLAB scripts and functions: http://www.mathworks.co.uk/matlabcentral/.
- R (http://www.r-project.org/) is a free environment for statistical computing and analysis. In addition to its built-in functionality, there are a large number of online repositories of R code. A good place to start is http://cran.r-project.org/.
- If you are writing C++ code then the following libraries may prove useful:
    - Boost (http://www.boost.org/) offers a large number of C++ libraries useful for scientific computing;
    - PETSc (http://www.mcs.anl.gov/petsc/) is a C++ library for performing large-scale matrix manipulation.
- Many libraries exist to extend the functionality of Python. Scientific Python (http://www.scipy.org/) adds numerous linear and nonlinear solvers and matplotlib (http://matplotlib.org/) adds high-quality graphical capabilities.
- Python Package Index (http://pypi.python.org), commonly known as 'PyPi', is a good place to find and share Python code.

Further reading

- W.B. Frakes and K. Kang. Software Reuse Research: Status and Future, IEEE Transactions on Software Engineering, 2005, 31(7), 529-536, http://doi.ieeecomputersociety.org/10.1109/TSE.2005.85.

- An interesting blog post on software reuse: http://www.infoq.com/articles/vijay-narayanan-software-reuse.

Rule 2: Develop a prototype first

Getting started

- Consider prototyping your code by implementing a simplified version first, and build up the functionality over several steps.
- While low-level languages such as C++ are undoubtedly faster in the long run, high-level languages such as those listed below offer useful debugging tools and other inbuilt functionality to help speed up prototype development.
- A short blog post guide to prototyping for scientist-programmers: http://www.programming4scientists.com/2008/09/08/prototyping-your-code/

Useful links

- MATLAB (http://www.mathworks.co.uk/): scientific computing environment and programing language based on principles of linear algebra.
- Gnu Octave (http://www.gnu.org/software/octave/): open source equivalent of MATLAB.
- Mathematica (http://www.wolfram.co.uk/mathematica/): environment for performing algebraic manipulation and algebraic solution of various types of equations.
- Maxima (http://maxima.sourceforge.net/): open source algebraic manipulation code.
- R (http://www.r-project.org/): language and environment for performing statistical computing and graphics.

Further reading

- J. Pitt-Francis and J. Whiteley. Guide to Scientific Computing in C++. Springer 2012. Chapter 1. (Note that many of the other rules presented in the paper appear as end of chapter ‘tips’ in this textbook.)

Rule 3: Make your code understandable to others (and yourself)

Getting started

- Making your code easily understood will help you, and others, maintain and debug it. This involves not just the code itself, but also descriptive comments. Consider what you would want to find if you were looking at someone else's code for the first time: your code should tell you *how* something is done and your comments should tell you *why*.
- Start with the basics: name and date each section of code you write (or consider using the date it was last edited). You'll surprise yourself at how useful a date can be.
- Using meaningful variable names greatly improves the readability of code, e.g. var_1 and var_2, versus BirthRate and DeathRate.
- Many languages have coding *conventions* that recommend a particular style for program code, covering comments, indentation, variable naming, etc. These vary between languages (you can found some examples at http://en.wikipedia.org/wiki/Comparison_of_programming_languages_(syntax)#Comments), but learning about these conventions can be useful for reading other people's source code and also for creating your own.

Useful links

- 'Writing Readable Source Code' article from the Software Sustainability Institute: http://software.ac.uk/resources/guides/writing-readable-source-code
- 'Writing Understandable Code' from Dr. Dobbs: http://www.drdobbs.com/writing-understandable-code/184414802
- Wikipedia article on source code comments, covering many languages and other uses of comments: http://en.wikipedia.org/wiki/Comment_(computer_programming)
- Wikipedia article reviewing coding conventions: http://en.wikipedia.org/wiki/Coding_conventions#Common_conventions
- Useful tools for generating documentation include Javadoc and Doxygen, which can produce cross-linked HTML from comments.
    - Doxygen (any language): http://www.doxygen.org/
    - Javadoc (Java): http://www.oracle.com/technetwork/java/javase/documentation/index-jsp-135444.html
    - Help files in MATLAB: http://www.mathworks.co.uk/help/matlab/matlab_prog/add-help-for-your-program.html
    - Pydoc (Python): http://docs.python.org/2/library/pydoc.html
- Tutorial for describing code using UML: http://www.tutorialspoint.com/uml/uml_building_blocks.htm

Further reading

- R.C. Martin. Clean code: a handbook of agile software craftsmanship. Prentice Hall, 2008.

- ‘Developing Maintainable Software’ from the Software Sustainability Institute: http://software.ac.uk/resources/guides/developing-maintainable-software (discusses the great idea of having your source code reviewed by your peers).
- C. Seiwald. The Seven Pillars of Pretty Code, EETimes 2003: http://eetimes.com/electronics-news/4196975/Seven-Pillars-of-Pretty-Code
- A. Oram and G. Wilson. Beautiful Code, Leading Programmers Explain How They Think. O'Reilly Media 2007.

Rule 4: Don't underestimate the complexity of your task

Getting started

There are some basic computational tools that will pay dividends if they are learnt early, some of which are more specific to coding practices, but some are also useful for the presentation of work:

- The Linux command line, as well as a command line text editor such as *emacs* or *vi*.
- Simple text file processing tools, such as *sed* and *awk*, and *grep* for searching for phrases in files (useful when you are debugging).
- Scripting languages such as *bash* (or more usefully *perl* or *python*).
- A build utility such as *make*, *CMake* or *SCons*.
- A job scheduler (if you don't want to be sat at your computer overnight!) such as *cron*.
- LaTeX for presenting mathematics in written documents.
- A literature reference (bibliography) manager.
- And of course the appropriate manual and help pages for the packages you are using.

Useful links

- List of useful Linux command lines: http://www.pixelbeat.org/cmdline.html.
- A useful introduction to LaTeX for beginners: http://www.andy-roberts.net/writing/latex.

Further reading

- The act of reviewing your code, looking for repeated functionality, and moving it into useful functions is commonly known as The Rule of Three. It was introduced in
  - M. Fowler, K. Beck, J. Brant, and W Opdyke. Refactoring: Improving the Design of Existing Code. Addison Wesley, 1999.
- There is a wealth of books about simple tools. An example is
  - C. Albing and J.P. Vossen. Bash Cookbook: Solutions and Examples for Bash Users. O'Reilly, 2007.

Rule 5: Understand the mathematical, numerical and computational methods underpinning your work

Getting started

The development of any computational method should involve a number of important issues:

- Ensure that your scientific approach is sound (modelling assumptions, suitability of analyses)
- Ensure that your chosen methods are accurate and stable
- Ensure that your implementation is efficient and bug free

While balancing these can be time-consuming and tricky, these issues underpin the ‘correctness’ of your approach and you should be aware of them in the development of your models and programs. For example, testing (Rule 8) can be useful for checking that you’ve implemented an algorithm correctly (or the library you’re using has) by validating results with some known solutions.

Useful links

- MIT Open Courseware in Numerical Analysis: http://ocw.mit.edu/courses/mathematics/18-03sc-differential-equations-fall-2011/unit-i-first-order-differential-equations/numerical-methods/
- Consider profiling to quantify the computational cost of different parts of your programs: http://en.wikipedia.org/wiki/Profiling_(computer_programming)
- The Software Carpentry lesson on algorithmic complexity: http://www.software-carpentry.org/4_0/invperc/tuning.html

Further reading

- K.W. Morton and D.F. Mayers. Numerical Solution of Partial Differential Equations. Cambridge University Press, 2005.
- G.H.Golub and C.F. Van Loan. Matrix Computations (3rd ed.). John Hopkins University Press, 1996.
- W.H. Press, S.A. Teukolsky, W.T. Vetterling, and B.P. Flannery. Numerical Recipes in C++: The Art of Scientific Computing (3rd ed.). Cambridge University Press, 2007.
- T. Cormen, C. Leiserson, R. Rivest, and C. Stein. Introduction to Algorithms (3rd ed.). MIT Press, 2009.
- D.E. Knuth. The Art of Computer Programming (multiple volumes). Addison Wesley, 2011.
- R. Gerber.The Software Optimization Cookbook: High-Performance Recipes for the Intel Architecture. Intel Press, 2002.
- A. Fog. Optimizing software in C++: http://www.agner.org/optimize/optimizing_cpp.pdf
- P. Getreuer. Writing Fast MATLAB code: http://www.mathworks.com/matlabcentral/fileexchange/5685

6: Use pictures: they really are worth a thousand words

Getting started

- Investing more time and effort in visualisation and graphics can enhance everything you do - from exploring data and debugging code/models all the way to conference presentations - so reassess the amount of effort and time you will invest. Find some galleries of useful visualisations/graphics and reconsider what you could achieve.
- From the outset think about what you want to achieve and what the visualizations purpose is. Who is it for? What is the key message? Writing a design brief (even quickly) can formalise these goals and provide a set of requirements that you can evaluate your visualisation by. Consider the audience and end-users at the start.
- Understand your workflow and what role your visualisations and graphics will have. Is it a throw-away graph to check for outliers in the data? Or will it really serve a purpose later on as well, e.g. in a presentation, software, a publication, or tutorial?
- Start with pen and paper before you start coding a visualisation and try different variations for the main axes of the figure, and the secondary axes. Understanding and thinking about the visual encodings you will use will save time later. If you can't say way you have made one design choice over another then go back to the drawing board.
- When implementing your design ensure that you write the visualisation as a function so that it can be re-used with any data set, and so any changes are easy to implement. For instance, if you are editing a paper and want to change the colour of some points you don't want to have to run all your models again. You should be able to supply all the data and code for a publication's figures as independent units.
- Treat you visualisations in the same way as you would text and test it out on your colleagues, friends and family. If it requires a PhD to read and understand your visualisations then you may need to go back to the drawing board. 'Cool' graphics serve a different purpose to 'informative' graphics so make sure that you are true to your design brief.

Useful links

Give your training in visualisation a reboot and read:

- A recent perspective on visualisation for biological data: S.I. O'Donoghue, A.C. Gavin, N. Gehlenborg, D.S. Goodsell, J.K. Heriche, C.B. Nielsen, C. North, A.J. Olson, J.B. Procter, D.W. Shattuck, T. Walter, and B. Wong. Visualizing biological data-now and in the future. Nature Methods. 2007. 7:S2-4. http://dx.doi.org/10.1038/nmeth.f.301
- A monthly column on design for scientific graphics: Wong, B. Points of view: Color coding. Nature Methods. 2010. 7, 573. http://dx.doi.org/10.1038/nmeth0810-573

Explore what tools are available and consider using a new tool to fit your goals:

- http://processing.org/ - '**Processing** is an open source programming language and environment for people who want to create images, animations, and interactions.'
- http://www.paraview.org/ - '**ParaView** is an open-source, multi-platform data analysis and visualization application. ParaView users can quickly build visualizations to analyze their data using qualitative and quantitative techniques. The data exploration can be done interactively in 3D or programmatically using ParaView's batch processing capabilities.'

- http://d3js.org/ - '**D3.js** is a JavaScript library for manipulating documents based on data. D3 helps you bring data to life using HTML, SVG and CSS. D3's emphasis on web standards gives you the full capabilities of modern browsers without tying yourself to a proprietary framework, combining powerful visualization components and a data-driven approach to DOM manipulation.'
- http://research.microsoft.com/en-us/um/cambridge/groups/science/tools/datasetviewer/datasetviewer.htm - '**DataSet Viewer** is a simple standalone menu-driven tool for quickly exploring and comparing time series, geographic distributions and other patterns within scientific data. DataSet Viewer combines selection, filtering and slicing tools, with various chart types (scatter plots, line graphs, heat maps, as well as tables), and geographic mapping (using Bing Maps). The resulting views can be exported as images or movies, or bundled into an interactive package that be shared with colleagues. '

Further reading

- A book about visualisation in practice and the 'Processing' visualisation language: B. Fry. Visualizing data. O'Reilly Media Inc, 2008. http://shop.oreilly.com/product/9780596514556.do
- A 'design' book about networks visualisations full of ideas: M. Lima. Visual Complexity: Mapping Patterns of Information. Princeton Academic Press, 2011. http://www.visualcomplexity.com/vc/
- An information visualisation blog - http://eagereyes.org/about
- Find some of Murrell's book on Graphics and plotting in R and code for his examples at http://www.stat.auckland.ac.nz/~paul/RGraphics/rgraphics.html
- Listen to the http://datastori.es/ podcast to hear about issues and solutions in data visualisation.
- If you can muster the cash, go to a visualisation conference: http://visweek.org/

Rule 7: Version control everything

Getting started

- Understand the capabilities of version control and how it can help you in your everyday work and how it can make collaborative work less painful. For that, we recommend watching this introductory video from the software carpentry course. http://software-carpentry.org/4_0/vc/intro.html
- Learn the jargon! Version control systems (VCS) are based on very simple ideas, but might seem overwhelming to the novice as they use very specific jargon such as commit, checkout, or cherry-pick. A good source to learn the words can be found in, 'A Visual guide to Version Control' (http://betterexplained.com/articles/a-visual-guide-to-version-control/). Also, be careful that the same terms can mean different things in different VCS! For example, 'merge' doesn't mean the same in git as it does in mercurial.
- Choose your version control system (even though there are several systems available which you may want to learn to use and alternate in the future, we advise you focus on one to start with). Before you can make this choice, it is crucial to understand the differences between a centralised and a distributed VCS (well explained here http://betterexplained.com/articles/intro-to-distributed-version-control-illustrated/). A good starting point for online repositories and open-source projects is http://github.com (and the techniques used here can later be transferred to other systems).
- Note that when writing documents in LaTeX using version control you could try start each sentence on a new line as this will make tracking changes much easier.

Useful links

Line-by-line version control tools - store the entire history of changes, and allows conflict resolution. Should be used for any type of code development or shared work.

- Subversion (SVN): http://subversion.apache.org/
  - Assembla: https://www.assembla.com/home a free SVN repository host.
- Git: www.github.com Good links to learn how to use Git are:
  - Git Immersion: 'A guided tour that walks through the fundamentals of git, inspired by the premise that to know a thing is to do it.': www.http://gitimmersion.com/
  - A Visual Git Reference: 'This page gives brief, visual reference for the most common commands in git.': http://marklodato.github.com/visual-git-guide/index-en.html
  - This memrise course is a very fun way to practice and memorise the key commands of Git: http://www.memrise.com/course/63157/git-commands-2/
- Mercurial: http://mercurial.selenic.com/

Also, even though not VCS as such (or with full VCS capabilities), the examples below are great for managing folders of documents, presentations, or visualisations that only you work on, or when you take turns with your collaborators.

- SkyDrive: http://www.skydrive.live.com
- Dropbox: http://www.dropbox.com
- Google Drive: https://drive.google.com

- Office 365: http://office365.microsoft.com/

Further reading

Version Control by Example: http://www.ericsink.com/vcbe/

Rule 8: Test everything

Getting started

- Test everything means test everything. Your code should be written in short logical parts (Rules 3 and 4). On this basis each single function should be tested separately and in combination with the others.
- Think about the oddest inputs and test all of them. Test all extreme cases. What would someone do just to make your program fail? Try to make your program fail!
- Think about writing your test cases before you actually start writing your program. At this time you will be way more motivated to write extensive test cases than afterwards. This approach will also help you to plan your program wisely.
- Find a tool for testing that you like to use (see links below) or design the testing environment by yourself. Either way test your code.

Useful links

- Overview about testing: http://software-carpentry.org/4_0/test/.
- Glossary for terms used in testing: http://www.origsoft.com/whitepapers/software-testing-glossary/glossary_of_terms.pdf.
- List of testing frameworks: http://en.wikipedia.org/wiki/List_of_unit_testing_frameworks.

Further reading

- Z. Merali. Why Scientific Programming Does Not Compute. Nature, 2010. 467:775–777. http://dx.doi.org/10.1038/467775a
- R. Sanders, D. Kelly. The Challenge of Testing Scientific Software. Proceedings Conference for the Association for Software Testing (CAST), Toronto, 2008, pages 30-36: http://www.cacr.caltech.edu/projects/danse/doc/kelly-sanders-01.pdf
- M. Feathers. Working Effectively with Legacy Code. Prentice Hall, 2004. http://rads.stackoverflow.com/amzn/click/0131177052
- B. Meyer. Seven Principles of Software Testing. Computer, 2008. vol.41, no.8, pages 99-101, http://dx.doi.org/10.1109/MC.2008.306

Rule 9: Share everything

Getting started

- Openness and sharing are now taken to be an important aspect of the scientific enterprise. For example, see the report on the uptake of open science: Science as an open enterprise The Royal Society Science Policy Centre report 02/2012: http://royalsociety.org/events/2012/science-open-enterprise/
- If open dissemination of your software/data is likely, addressing this from the beginning will save time later. For example, place your outputs in a public version controlled repository like GitHub (c.f. rule 7).
- A recent paper focusing on developing open source software: A. Prlić, J.B. Procter. Ten Simple Rules for the Open Development of Scientific Software. PLoS Comput Biol 2012 8(12): e1002802. http://dx.doi.org/10.1371/journal.pcbi.1002802

Useful links

- A blog on developing and releasing free and open source software: http://www.openscience.org/.
- An open repository for the deposition and sharing of protocols for scientific research: http://www.nature.com/protocolexchange/.
- Dryad: an international repository of data underlying peer-reviewed articles in the basic and applied biosciences: http://datadryad.org/.
- The Open Source Initiative: advocate of open source software: http://opensource.org/.
- The Open Data Institute: http://www.theodi.org/.
- Figshare: a repository where users can make all of their research outputs, in any file format, available in a citable, sharable and discoverable manner: http://figshare.com.

Further reading

- Links to advice on copyright issues:
  - http://toolkit.vph-noe.eu/component/docman/doc_download/1-g07-toolkit-licensing-guideline-v10
  - http://www.oss-watch.ac.uk/resources/iprguide
- D. Ince, L. Hatton, and J. Graham-Cumming. The case for open computer programs. Nature 2012. Volume 482 pages 485-488. http://dx.doi.org/doi:10.1038/nature10836
